# Association between lumbopelvic motion and muscle activation in patients with non-specific low back pain during forward bending task: A cross-sectional study

**DOI:** 10.1142/S1013702520500043

**Published:** 2019-12-30

**Authors:** Peemongkon Wattananon, Komsak Sinsurin, Sirikarn Somprasong

**Affiliations:** 1 Motor Control and Neural Plasticity Laboratory, Faculty of Physical Therapy, Mahidol University, Nakhon Pathom, Thailand; 2 Biomechanics and Sports Research Unit, Faculty of Physical Therapy, Mahidol University, Nakhon Pathom, Thailand; 3 Faculty of Physical Therapy, Mahidol University, Nakhon Pathom, Thailand; peemongkon.wat@mahidol.ac.th

**Keywords:** Lumbopelvic movement pattern, lumbopelvic muscle activation pattern, non-specific low back pain

## Abstract

**Background:**

Evidence suggests patients with non-specific low back pain (NSLBP) have altered lumbar and pelvic movement patterns. These changes could be associated with altered patterns of muscle activation.

**Objective:**

The study aimed to determine: (1) differences in the relative contributions and velocity of lumbar and pelvic movements between people with and without NSLBP, (2) the differences in lumbopelvic muscle activation patterns between people with and without NSLBP, and (3) the association between lumbar and pelvic movements and lumbopelvic muscle activation patterns.

**Methods:**

Subjects (8 healthy individuals and 8 patients with NSLBP) performed 2 sets of 3 repetitions of active forward bending, while motion and muscle activity data were collected simultaneously. Data derived were lumbar and pelvic ranges of motion and velocity, and ipsilateral and contralateral lumbopelvic muscle activities (internal oblique/transverse abdominis (IO/TA), lumbar multifidus (LM), erector spinae (ES) and gluteus maximus (GM) muscles).

**Results:**

Lumbar and pelvic motions showed trends, but exceeded 95% confidence minimal detectable difference (MDD${}_{95})$, for greater pelvic motion (p=0.06), less lumbar motion (p=0.23) among patients with NSLBP. Significantly less activity was observed in the GM muscles bilaterally (p<0.05) in the NSLBP group. A significant association (r=−0.8, p=0.02) was found between ipsilateral ES muscle activity and lumbar motion, while moderate, but statistically non-significant associations, were found between GM muscle activity bilaterally and lumbar velocity (ipsilateral: r=−0.6, p=0.14; contralateral: r=−0.6, p=0.16) in the NSLBP group.

**Conclusion:**

Findings indicated patients had greater pelvic contribution, but less lumbar contribution which was associated with less activation of the GM bilaterally.

## Introduction

Low back pain is a common health problem in many countries with high prevalence and recurrence rates.^1,2^ Non-specific low back pain (NSLBP) is assigned when a recognizable or known specific pathology cannot be identified.^3^ NSLBP is accountable for approximately 85% of low back pain.^4^ Inappropriate management of low back pain can result in perpetuation and recurring low back episodes which can further cause financial burden for health care systems.^5-7^

Lack of understanding of underlying low back pain mechanism is one contributing factor responsible for such high recurrence rates.^8,9^ Current research studies have demonstrated that patients with NSLBP have altered lumbar and pelvic movement patterns including relative contribution and velocity during an active forward bend.^10-14^ One systematic review indicated altered lumbar and pelvic contribution among patients with NSLBP during the active forward bend.^11^ This systematic review also demonstrated consistent findings for reduced lumbar velocity during this task.^11^ Researchers interpreted slow lumbar velocity as an indicator of coping strategy to minimize excessive lumbar motion.^15-17^ However, results from the lumbar segment alone may be insufficient to describe this phenomenon. Therefore, investigating the relative velocity of the lumbar spine and pelvis should support the interpretation in which patients with NSLBP use lumbar coping strategy to minimize excessive lumbar motion.

In addition, clinicians have suggested that altered lumbar and pelvic relative contribution and velocity among patients with NSLBP are associated with lumbopelvic muscle activation deficits.^18-21^ Lumbopelvic muscles include the bilateral internal oblique/transverse abdominis (IO/TA), lumbar multifidus (LM), lumbar erector spinae (ES), and gluteus maximus (GM) muscles. They have been proposed as key contributors to provide dynamic stability during functional movement.^6,22-24^ These functionally-impaired muscles should be responsible for changes in lumbar and pelvic movement patterns. However, the association between underlying lumbar and pelvic movement patterns and lumbopelvic muscle activation patterns during active forward bending among patients with NSLBP has not been systematically investigated.

Therefore, this study aimed to: (1) determine the difference in lumbar and pelvic movement patterns (relative contribution and velocity) between healthy individuals (CON) and patients with NSLBP (LBP), (2) determine the extent of differences in lumbopelvic muscle activation patterns (IO/TA, LM, ES, and GM) between CON and LBP, and (3) determine association between lumbar and pelvic movement patterns and lumbopelvic muscle activation patterns. We hypothesized that patients with NSLBP would have altered lumbar and pelvic relative contribution and velocity, as well as lumbopelvic muscle activation patterns. We also further hypothesized that associations would be found between lumbar and pelvic movement patterns and lumbopelvic muscle activation patterns. Enhanced knowledge resulting from this study would provide a significant step toward investigating underlying neuromuscular mechanisms, and the ability of exercise intervention to restore lumbar and pelvic movement patterns and lumbopelvic muscle activation patterns. The long term outcomes of this research could help improve physical therapy interventions specific to patients with NSLBP; thereby, optimizing clinical outcomes and preventing recurring symptoms.

## Methods

### Subjects

Eight patients with NSLBP between the ages of 21 and 65, and 8 age-, sex-, and BMI-matched healthy individuals were recruited from the University Physical Therapy Clinic. Additional inclusion criteria for patients with NSLBP included current episode of back pain less than three months causing them to seek medical or physical therapy intervention and not receiving any intervention involving the core stability in the last six months. Subjects were excluded if they presented clinical signs of systemic disease, definitive neurologic signs including weakness or numbness in the lower extremity, previous spinal surgery, osteoporosis, severe spinal stenosis, or inflammatory joint disease, pregnancy, any lower extremity condition that would potentially alter trunk movement in standing, vestibular dysfunction, extreme psychosocial involvement, and BMI greater than 30 kg/m^2^. This study constituted one part of the intervention with a pre-specified sample size; therefore, we did not perform sample size calculation. However, sample size requirements were derived for future replication of this study.

### Instrumentation and measures

Electromagnetic tracking system (3D Guidance trakSTAR, Ascension Technology Corp., Vermont, USA) was used for motion data collection. Criteria-related validity with known quantity has been reported by the manufacturer. The coefficient of multiple determination demonstrated excellent $\left(R^{2}=0.98\right)$ test–retest reliability of this system. Three sensors were attached to the subjects at the following landmarks: (1) the right femur (bony prominence of the right femoral lateral epicondyle); (2) the pelvis (over the spinous process of S2); and (3) the lumbar spine (over the spinous process of L1). These sensor placements were based upon recommendations of the International Society of Biomechanics.^25^ This tracking system collected kinematic data at 100 Hz. Related work has demonstrated kinematics in conjunction with a dynamic systems approach that could be used to quantify movement patterns that represent clinically observed aberrant movement patterns.^14^

Electromyography (EMG) (TeleMyo 2400R G2, Noraxon Inc., Arizona, USA; common mode rejection ratio > 100 dB, input impedance > 100 MOhm, 500 gain) with pre-amplified bipolar electrodes (Kendall Medi-Trace 100, Kendall Inc.; Al/AgCl, disc-shaped, 1 cm diameter) was used to collect muscle activity from bilateral IO/TA, LM, ES, and GM. Skin was lightly abraded using abrasive paper and cleaned using cotton with alcohol to lower the skin impedance. IO/TA electrodes were placed at 2 cm medial to ASIS and on the inguinal line. LM electrodes were placed at 2 cm lateral to the L5 spinous process. ES electrodes were placed at 3 cm lateral to the L1 spinous process. GM electrodes were placed at midpoint between the greater trochanter and the last sacral vertebrae.^24^ Electrodes were placed parallel to the muscle fibers with an inter-electrode distance of 2 cm. Analog EMG data included bandpass-filtered (10–1500 Hz), and differentially amplified to +/−5 V.

### Procedures

This study employed a cross-sectional design. The institutional review board approval from the university was obtained (COA No. 2015/050.3004) before collecting data. Data were collected at the university laboratory (Motor Control and Neural Plasticity Laboratory) from August 2016 to October 2017. Each subject underwent the written informed consent process before providing data. Electromagnetic sensors and surface EMG electrodes were attached to the subjects’ body landmarks. Subjects were instructed to perform a modified Sorensen test at submaximal level (15% of body weight) to derive bilateral LM and ES reference voluntary contraction (RVC). We used this submaximal level to avoid aggravating pain, which could change muscle activation patterns. In addition, subjects were asked to perform the maximal contraction of the hip extension in a prone position with 90^∘^ knee flexion position, and maximal abdominal hollowing in a crook lying position to derive RVC for GM and IO/TA, respectively.^26,27^ These RVC were further used to normalize EMG data for each muscle group. Subjects were instructed to perform 2 sets of 3 consecutive repetitions of forward bending movement. The instruction was “please stand relaxed with equal weight on both legs, and then try to reach toward the floor as far as you can at your comfortable pace and return to starting position”. Motion and EMG data were simultaneously recorded.

### Data reduction

Data reduction was performed using a customary LabVIEW programming (National Instruments Corp.). Motion data were converted to segment angular rotations using Euler’s angle in Cardan sequence (x, y, and z). Segment angular rotations included lumbopelvic motion (lumbar sensor in respect to femur sensor), lumbar motion (lumbar sensor in respect to pelvic sensor), and pelvic motion (pelvic sensor in respect to femur sensor). These data were filtered using a dual-pass Butterworth (2nd order low pass frequency at 5 Hz). Lumbar and pelvic ranges of motion and velocity were obtained. Maximal range of motion, time to maximal range of motion, maximal angular velocity, and time to maximal angular velocity for lumbar spine (MaxR_*L*_, TtoMaxR_*L*_, MaxV_*L*_, and TtoMaxV_*L*_), and pelvis (MaxR_*P*_, TtoMaxR_*P*_, MaxV_*P*_, and TtoMaxV_LP_) for each repetition were derived. Averaged motion parameters across the first 3 and the last 3 repetitions were used to establish test–retest reliability and 95% confidence minimal detectable difference (MDD${}_{95}$). All kinematics were analyzed and reported in the sagittal plane.

EMG data were filtered using independent component analysis to remove heart rate artifacts. Heart rate filtered EMG were further filtered using a band pass filter (2nd order Butterworth high pass at 20 Hz and low pass at 400 Hz) and a band stop filter (2nd order Butterworth at 50 Hz). These data were full-wave rectified and underwent data smoothing using root mean square (RMS) with a time constant of 50 ms. RVC data between 2 and 4 s was used to normalize muscle activity during the forward bending task. However, our preliminary data analysis demonstrated that pain location among patients with NSLBP could change muscle activation patterns; therefore, we separately analyzed muscle groups ipsilateral and contralateral to the pain location for the main analysis. Ipsilateral (I) and contralateral (C) peak EMG amplitudes for each muscle (IIO/TA, CIO/TA, ILM, CLM, IES, CES, IGM, and CGM) were derived. Similar to motion data, averaged EMG parameters across the first and last three repetitions were used to establish test–retest reliability and MDD${}_{95}$.

### Statistical analysis

Statistical analysis was performed using SPSS Software, Version 21.0 (IBM Corp., New York, USA). Intra-class correlation coefficients (ICC${}_{3,3}$) were used to establish test–retest reliability of motion and EMG parameters, and MDD${}_{95}$ were calculated. The normality test was performed using Kolmogorov–Smirnov goodness-of-fit test. When demographic data were normally distributed, the independent t-test was used. Otherwise, the Mann–Whitney’s U test was used. The Chi-square test was used to compare sex. For motion data, a mixed design ANOVA with *post-hoc* pairwise comparisons (Bonferroni adjustment) was used for normally distributed data, while non-parametric statistics was used for non-normally distributed data. For EMG data, independent t-tests were used when data were normally distributed; otherwise, Mann– Whitney’s U tests were used instead. To determine the association between motion and muscle activity, Pearson’s correlations were used when data were normally distributed, while Spearman’s rank tests were used when data were non-normally distributed. Confidence level (α) was set at 0.05.

## Results

Demographic data (Table 1) demonstrated no significant difference (p>0.05) in age, sex, BMI, as well as lumbar and pelvic ranges of motion and velocities between CON and LBP groups. Intra-class correlation coefficient (ICC${}_{3,3})$ demonstrated excellent test–retest reliability (ICC${}_{3,3}$ ranged between 0.90 and 1.00) of EMG and motion parameters, and a 95% confidence MDD${}_{95}$ was established (Appendix).

**Table 1.** Demographic data.

Parameter	CON (N=8)	LBP (N=8)	p-value
Age ± SD (years)	27.7 ± 5.0	29.4 ± 5.2	0.54
Sex (% female)	42.9	42.9	1.00
BMI (kg/m^2^)	22.1 ± 2.3	24.5 ± 2.2	0.08
MaxR_LP_ ± SD (degrees)	98.9 ± 14.4	104.4 ± 12.1	0.42
MaxV_LP_ ± SD (degrees/sec)	102.2 ± 29.0	98.8 ± 44.4	0.86
NPRS (out of 10)	N/A	5.7 ± 1.9	N/A
ODI (percent)	N/A	19.7 ± 7.5	N/A

*Notes*: CON = Healthy controls, LBP = Low back pain, SD = Standard deviation, BMI = Body mass index, MaxR${}_{\text{LP}}=\text{Lumbopelvic}$ maximal range of motion, MaxV${}_{\text{LP}}=\text{Lumbopelvic}$ maximal velocity, NPRS = Numeric pain rating scale, ODI = Oswestry disability index, N/A = Not applicable.

Table 2 demonstrates results from mixed ANOVA with *post-hoc* pairwise comparisons. Results demonstrated a trend in the interaction effect of Group * Segment (p=0.05), and a significant main effect of Segment (p<0.001). *Post-hoc* comparisons showed that the LBP group exhibited a trend of greater MaxR_*P*_ compared with the CON group (p=0.06). The MaxR_*L*_ was similar between the LBP and CON groups (p=0.23). No significant interaction effect was observed for velocity. The velocity result demonstrated only a significant main effect of Segment (p=0.03). *Post-hoc* simple within-group comparisons demonstrated significantly greater MaxV_*P*_ compared with MaxV_*L*_ (p=0.03) in the LBP group only.

**Table 2.** Interaction and main effects from mixed ANOVAs, as well as between groups and within group post-hoc pairwise comparisons.

Kinematics	Mixed ANOVA		F value	p-value	$\eta^{2}$		Parameters	CON (Mean ± SD)	LBP (Mean ± SD)	Between-group mean diff	MDD${}_{95}$	p-value
ROM	Interaction effect	Group × Segment	4.44	0.05	0.24	Post-hoc comparison	MaxR_*L*_ (degrees)	42.5 ± 11.3	36.0 ± 9.6	6.53*	2.29	0.23
							MaxR_*P*_ (degrees)	56.4 ± 12.6	68.4 ± 10.5	−12.03*	4.22	0.06
	Main effect	Group	0.68	0.42	0.05		Within-group mean diff	−13.9	−32.4			
		Segment	27.70	<0.001	0.66		p-value	0.04	<0.001			
Velocity	Interaction effect	Group × Segment	0.74	0.40	0.05	Post-hoc comparison	MaxV_*L*_ (degrees/second)	58.1 ± 13.0	53.3 ± 23.4	4.70	7.10	0.63
							MaxV_*P*_ (degrees/second)	67.2 ± 24.8	72.3 ± 34.7	−5.10	13.23	0.74
	Main effect	Group	<0.001	0.99	<0.001		Within-group mean diff	−9.1	−18.9			
		Segment	6.02	0.03	0.30		p-value	0.28	0.03			

*Notes*: MaxR${}_{L}=\text{Lumbar}$ maximal range of motion, MaxR${}_{P}=\text{Pelvic}$ maximal range of motion, MaxV${}_{L}=\text{Lumbar}$ maximal velocity, MaxV${}_{P}=\text{Pelvic}$ maximal velocity, CON = Healthy controls, LBP = Low back pain, SD = Standard deviation, Mean diff = Mean difference, MDD${}_{95}=95$% confidence minimal detectable difference. ∗=Exceed 95% confidence minimal detectable difference.

Non-parametric Mann–Whitney’s U tests for EMG data showed significant lower activation of both IGM and CGM in the LBP group compared with CON (p=0.02 and 0.04, respectively); however, only median IGM exceeded MDD${}_{95}$ (Table 3).

**Table 3.** Lumbopelvic muscle activation pattern comparison between groups.

Muscle	CON Median [ICR]	LBP Median [ICR]	Median diff	MDD${}_{95}$	p-value
IIO/TA	54.4 [14.5, 85.6]	21.5 [15.4, 68.6]	32.9	34.9	0.65
CIO/TA	53.8 [7.0, 76.4]	24.5 [14.0, 72.6]	29.3	29.4	0.96
ILM	77.6 [27.8, 179.1]	24.6 [20.0, 83.2]	53.0	274.7	0.33
CLM	51.6 [27.3, 84.5]	27.1 [24.1, 46.6]	24.5	31.9	0.44
IES	54.2 [37.6, 367.6]	62.2 [18.6, 265.1]	−8.0	80.2	0.57
CES	175.3 [31.9, 316.4]	54.4 [22.2, 139.0]	121.0	95.9	0.51
IGM	59.6 [31.0, 107.3]	21.3 [12.2, 37.7]	38.4*	21.1	0.02
CGM	39.6 [30.7, 81.6]	18.6 [13.8, 30.6]	21.0	76.6	0.04

*Notes*: I = Ipsilateral, C = Contralateral, IO/TA = Internal oblique/transverse abdominis, LM = Lumbar multifidus, ES = Erector spinae, GM = Gluteus maximus, CON = Healthy controls, LBP = Low back pain, SD = Standard deviation, ICR = Interquartile range, Mean diff = Mean difference, Median diff = Median difference, MDD${}_{95}=95$% confidence minimal detectable difference. ∗=Exceed 95% confidence minimal detectable difference.

Correlations between lumbar and pelvic movement patterns and muscle activation patterns were determined using Spearman’s rank test (Table 4). Correlation results in the LBP group demonstrated that a strongly significant negative association (r=−0.8, p=0.02 was found between IES and MaxR_*L*_.

**Table 4.** Correlation between lumbopelvic movement pattern (spatial and temporal) and lumbopelvic muscle activation patterns based on all subjects.

		Spatial parameter				Temporal parameter			
Group	Muscle	MaxR_*L*_	MaxV_*L*_	MaxR_*P*_	MaxV_*P*_	TtoMaxR_*L*_	TtoMaxR_*P*_	TtoMaxV_*L*_	TtoMaxV_*P*_
LBP	IIO/TA	0.3	0.1	−0.3	0.1	0.2	0.2	0.2	0.1
	CIO/TA	−0.3	0.1	0.2	0.4	−0.3	−0.1	−0.1	−0.2
	ILM	0	−0.2	−0.5	−0.2	0.3	0.1	0	0.2
	CLM	−0.6*	−0.1	0.2	0.2	−0.4	−0.3	−0.4	−0.2
	IES	−0.8**	−0.1	0.1	0.4	−0.3	−0.2	−0.2	−0.1
	CES	0.3	0.1	−0.4	−0.2	0.1	−0.2	−0.3	0
	IGM	−0.1	−0.6*	0	−0.4	0.5*	0.6*	0.4	0.6
	CGM	−0.4	−0.6*	−0.5	−0.4	0.2	0	−0.1	0.2
CON	IIO/TA	−0.2	−0.8**	0.1	0	0.1	−0.1	−0.1	0
	CIO/TA	−0.4	−0.5*	0	0	0	−0.1	−0.1	−0.2
	ILM	0.1	−1.0**	0.1	−0.1	0.1	0	−0.2	0.1
	CLM	−0.2	−0.6*	0.2	0.3	−0.2	−0.4	−0.5*	−0.2
	IES	−0.2	−0.5*	0.3	0.2	−0.3	−0.5*	−0.4	0
	CES	−0.4	−0.7*	0	0.1	−0.1	−0.2	−0.3	0
	IGM	0.1	−0.8**	0	−0.1	0	−0.2	−0.3	0.3
	CGM	0.4	−0.8**	0	−0.2	0.3	0.1	−0.1	0.4

*Notes*: LBP = Low back pain group, CON = Healthy control group, I = Ipsilateral, C = Contralateral, IO/TA = Internal oblique/transverse abdominis, LM = Lumbar multifidus, ES = Erector spinae, GM = Gluteus maximus, L = Lumbar spine, P = Pelvis, MaxR = Maximal range of motion, TtoMaxR = Time to maximal range of motion, MaxV = Maximal velocity, TtoMaxV = Time to maximal velocity. *Notes*: Statistical analysis was performed using Spearman’ rank test. ∗∗=Strong association (r>0.70) with statistical significance (p<0.05). ∗=Moderate association r between 0.50 and 0.69), but not statistical significance (p>0.05).

When analyzing the CON group (Table 4), strongly significant negative associations were found between IIO/TA and MaxV_*L*_ (r=−0.8, p=0.02), ILM and MaxV_*L*_ (r=−1.0, p<0.001), IGM and MaxV_*L*_ (r=−0.8, p=0.02), and CGM and MaxV_*L*_ (r=−0.8, p=0.01).

## Discussion

Demographic data indicated both groups were comparable, and performed the same task. Test–retest reliability of EMG and motion parameters were excellent indicating that subjects consistently performed the active forward bend task and our measurement was reliable, allowing us to confidently interpret our data as a true difference between group comparisons when difference exceeds MDD${}_{95}$.^28^

We have simultaneously collected lumbar and pelvic motion and lumbopelvic muscle activity data in this study. This allows us to comprehensively explain altered lumbar and pelvic relative contributions, and changes in lumbopelvic muscle activation responsible for those changes in relative contribution during an active forward bend.

Although healthy individuals and patients with NSLBP performed the same active forward bend task, we found that patients with NSLBP tended to use lower lumbar contribution, but significantly greater pelvic contribution, than those among healthy individuals. Our motion results were consistent with several lumbar and pelvic motion studies in which they found similar changes in lumbar and pelvic contributions.^10-14^ In addition, within-group comparisons suggested that patients with NSLBP obviously used pelvic dominate movement patterns, while healthy individuals used shared patterns between lumbar spine and pelvis during active forward bend.^14^

To our knowledge, no study has investigated the relative velocity of the lumbar spine and pelvis during the active forward bend. Our findings suggested that patients with NSLBP may attempt to compensate slow lumbar motion by increased pelvic velocity. In other words, slower lumbar motion among patients with NSLBP may indicate that they attempted to minimize lumbar motion to prevent excessive lumbar motion.^15-17^

Findings in muscle activation patterns suggested that patients with NSLBP had lower muscle activation of the bilateral GM muscles. Our results were consistent with one related study in which they found that GM muscles were more fatigable among patients with NSLBP.^23^ Lower bilateral GM muscle activation could alter body mechanics during trunk flexion and extension, particularly load transfer in the lumbar spine.^23^ This could further lead to stress on the lumbar region; thereby, developing a low back symptom.^17,29^

Patients with NSLBP demonstrated coping strategy by increased ipsilateral ES muscle activation to minimize this shear force on the lumbar spine,^17^ which was supported by a strong negative association between the ipsilateral ES muscle and maximal lumbar range of motion.

Lumbar maximal velocity representing trunk neuromuscular control on the lumbar motion^14^ seemed to be modulated by all lumbopelvic muscles evident by a moderate to strong negative association between each lumbopelvic muscle and maximal lumbar velocity among healthy individuals. Notably, the bilateral GM muscles might be key muscles that need to be considered when treating patients with NSLBP. Based upon our findings, the bilateral GM muscles were strongly associated with lumbar maximal velocity among both patients with NSLBP and healthy individuals indicating the importance of these muscles to control lumbar spine motion. Inadequate activation of the bilateral GM muscles could cause altered lumbar spine control, as well as excessive hip motion. This would in turn increase stress on the lumbar spine leading to low back symptoms.^17,29^

Our findings suggest that clinicians should focus on the lumbar and pelvic contribution by restoring lumbopelvic muscle activation patterns among patients with NSLBP. Specifically, interventions should be designed to restore GM muscle activation to prevent excessive pelvic motion. Control of lumbar spine motion is also a key factor for managing patients with NSLBP. Restoring lumbar control would provide dynamic stability, and could be achieved by activating the bilateral lumbopelvic muscles. Therefore, excessive load on the lumbar spine would be minimized. This would also help to prevent recurring episodes of low back symptoms.

The findings of this study should be considered in light of the following limitations. This study was part of an intervention study that pre-specified a sample size of 16 (8 subjects per group). Therefore, findings associated between lumbar and pelvic motions and lumbopelvic muscle activation in our study tended to be under-powered. A minimum sample size of 24 would be required to detect significant associations using a calculated effect size, 80% power and confidence level of 0.05. Our study design, employed sub-maximal voluntary isometric contractions of the back muscles (the bilateral lumbar multifidus and ES muscles) to avoid pain exacerbation, while we used maximal contractions for other muscles, which would have limited the within-group comparisons. Therefore, we were unable to compare the level of activation across the lumbopelvic muscles. In addition, future studies may incorporate our findings to refine the intervention that addresses the lumbar and pelvic contributions, as well as muscle activation patterns. An intervention study would provide evidence to support whether changes in those motions and muscle activation would be effective in managing patients with NSLBP and minimize recurrence rate.

Our study concurrently investigated lumbar and pelvic motions and lumbopelvic muscle activation to enable the comprehensive analysis of underlying mechanisms during active forward bend. We found the difference in lumbar and pelvic contributions between patients with NSLBP and healthy individuals even though they were performing the same task. Patients with NSLBP had less activation of the bilateral GM muscle associated with lumbar maximal velocity. These findings suggested that contributions of the lumbar spine and pelvis, as well as GM muscle activation should be considered for managing patients with NSLBP.

## Acknowledgments

We would like to thank Motor Control and Neural Plasticity Laboratory, Mahidol University for providing data collection space and equipment. We would also like to thank Ms. Tanatta Chichakan and Mr. Pisit Suwanimit for helping us in collecting data. We also wish to thank all the subjects who participated in this study.

## Conflict of Interest

The authors declare that they have no conflict of interest.

## Funding/Support

This study was funded by the Thailand Research Fund (Grant No. TRG5880133, 2015).

## Author Contributions

PW substantially contributed to the concept, research design, data collection and analysis, paper preparation and edition. KS and SS have significantly contributed to data analysis and revising the paper. All authors read and approved the final paper.

## Appendix A

**Table A.1.** Test–retest reliability, standard error of measurement, and 95% confidence minimal detectable difference for each parameter.

Variable	ICC${}_{3,3}$	lower ICC	upper ICC	SEM	MDD${}_{95}$
IIO/TA	1.00	0.99	1.00	0.13	0.35
CIO/TA	0.99	0.96	1.00	0.11	0.29
ILM	0.92	0.77	0.97	0.99	2.75
CLM	1.00	0.99	1.00	0.12	0.32
IES	0.91	0.74	0.97	0.29	0.80
CES	1.00	0.99	1.00	0.35	0.96
IGM	0.96	0.88	0.99	0.08	0.21
CGM	0.90	0.72	0.97	0.28	0.77
MaxR_*L*_	0.99	0.98	1.00	0.83	2.29
MaxR_*P*_	0.99	0.96	1.00	1.52	4.23
MaxR_LP_	0.99	0.96	1.00	1.57	4.35
TtoMaxR_*L*_	0.96	0.89	0.99	17.29	47.92
TtoMaxR_*P*_	0.96	0.89	0.99	17.92	49.66
TtoMaxR_LP_	0.97	0.92	0.99	16.61	46.03
MaxV_*L*_	0.98	0.95	0.99	0.03	0.07
MaxV_*P*_	0.97	0.93	0.99	0.05	0.13
MaxV_LP_	0.99	0.96	1.00	0.05	0.12
TtoMaxV_*L*_	0.97	0.91	0.99	8.68	24.06
TtoMaxV_*P*_	0.96	0.87	0.99	14.73	40.83
TtoMaxV_LP_	0.97	0.93	0.99	8.44	23.38

*Notes*: ICC = Intra-class correlation coefficient, SEM = Standard error of measurement, MDD${}_{95}=95$% confidence minimal detectable difference, I = Ipsilateral, C = Contralateral, IO/TA = Internal oblique/transverse abdominis, LM = Lumbar multifidus, ES = Erector spinae, GM = Gluteus maximus, L = Lumbar spine, P = Pelvis, LP = Lumbopelvic, MaxR = Maximal range of motion, TtoMaxR = Time to maximal range of motion, MaxV = Maximal velocity and TtoMaxV = Time to maximal velocity.
